# An Unusual Congenital Anomaly in Fanconi Aplastic Anemia: Congenital Lobar Emphysema

**DOI:** 10.4274/tjh.2015.0384

**Published:** 2016-08-19

**Authors:** Ali Fettah, Gökçe Pınar Reis, Soner Sertan Kara, Tekin Aksu, Afak Durur Karakaya, Mahmut Subaşı, Atilla Çayır

**Affiliations:** 1 Erzurum Regional Training and Research Hospital, Clinic of Pediatric Hematology, Erzurum, Turkey; 2 Erzurum Regional Training and Research Hospital Clinic of Pediatric Infectious Disease, Erzurum, Turkey; 3 Dr. Abdurrahman Yurtaslan Oncology Training and Research Hospital, Clinic of Pediatric Hematology, Ankara, Turkey; 4 Erzurum Regional Training and Research Hospital, Clinic of Radiology, Erzurum, Turkey; 5 Erzurum Regional Training and Research Hospital, Clinic of Thoracic Surgery, Erzurum, Turkey; 6 Erzurum Regional Training and Research Hospital, Clinic of Endocrinology, Erzurum, Turkey

**Keywords:** Fanconi, Anemia, Congenital lobar emphysema

A 7-year-old girl who presented with epistaxis was examined due to pancytopenia. Her medical history revealed that she had respiratory distress in the neonatal period. She was born to a second-degree consanguineous marriage. Physical examination revealed short stature, microcephaly, microphthalmia, and hypo/hyperpigmented lesions on the trunk and extremities. She did not have tachypnea, but she had decreased breathing sounds in the left lung. A laboratory work-up revealed hemoglobin of 5.4 g/dL, mean corpuscular volume of 103/fL, leukocyte count of 2.7x109/L, and thrombocyte count of 11x109/L. A chromosomal breakage test with diepoxybutane was compatible with Fanconi anemia (FA). Posteroanterior chest X-ray showed hyperinflation of the left lung (Figure 1). Chest computed tomography revealed emphysematous changes in the upper part of the left lung, compatible with congenital lobar emphysema (Figure 2).

FA is a rare autosomal recessive disorder and presents with numerous organ abnormalities, progressive cytopenia, and susceptibility to several malignancies [1,2]. Although absent lung lobes and abnormal pulmonary drainage have been reported [3], congenital lobar emphysema has not been presented as an accompanying pathology with FA. It is striking that the patient had no prominent respiratory symptoms since the newborn period. Congenital lobar emphysema’s association with FA has not been reported previously and it could be in coexistence or have an association with FA.

## Ethics

Informed Consent: It was taken.

## Figures and Tables

**Figure 1:**
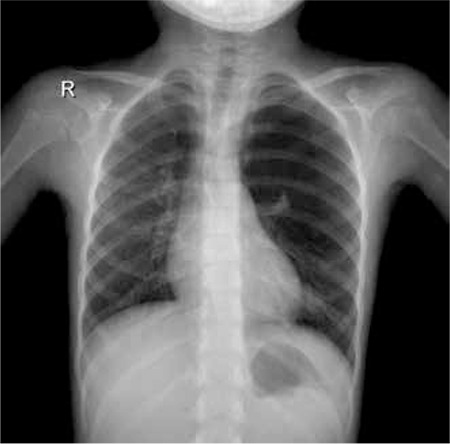
Posterior anterior lung radiography imaging of the patient.

**Figure 2:**
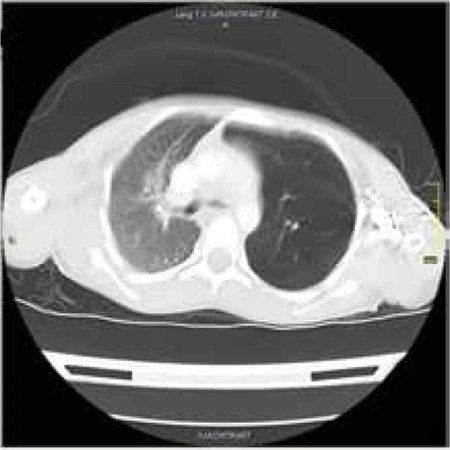
Computerized tomography imaging of the patient.
